# An analysis of the vitamin D overtesting in a tertiary healthcare centre

**DOI:** 10.11613/BM.2022.020701

**Published:** 2022-04-15

**Authors:** Merica Aralica, Vesna Šupak Smolčić, Tamara Turk Wensveen, Snježana Hrabrić Vlah, Mihael Selar, Lidija Bilić Zulle

**Affiliations:** 1 Clinical Department of Laboratory Diagnostics, Clinical Hospital Centre Rijeka, Rijeka, Croatia; 2 Department of Medical Informatics, Rijeka University School of Medicine, Rijeka, Croatia; 3 Center for Diabetes, Endocrinology and Cardiometabolism, Thallassotherapia, Opatija, Croatia; 4 Department of Endocrinology, Diabetes and Metabolic Disorders, Clinical Hospital Centre, Rijeka, Croatia; 5 Department of Internal Medicine, Faculty of Medicine, University of Rijeka, Rijeka, Croatia; 6 Department of Medical Biochemistry and Laboratory Medicine, General Hospital Pula, Pula, Croatia

**Keywords:** vitamin D testing, 25(OH) D, guideline, overtesting, financial burden

## Abstract

**Introduction:**

Vitamin D testing is excessively used in clinical practice, despite of the clinical guidelines statements against population screening for vitamin D deficiency. This study aimed to assess an annual number of performed 25-hydroxy vitamin D (25(OH)D) tests that were unsupported by the national guidelines for prevention, detection and therapy of vitamin D deficiency in adults and to calculate associated financial burden for the publicly funded healthcare.

**Materials and methods:**

A representative sample of requested 25(OH)D tests in 2018 (N = 474) was formed after selection and randomisation of data set (N = 5298) collected from the laboratory information system database of the Clinical Department for Laboratory Diagnostics, the Clinical Hospital Centre Rijeka. Records were classified in two groups depending on associated medical condition(s) according to the national guidelines. An annual cost of the total and group specific vitamin D testing was calculated on the base of a single test price reimbursed by the Croatian Healthcare Insurance Fund (CHIF).

**Results:**

Medical conditions with high-risk for vitamin D deficiency were detected in 43% (206/474) of vitamin D requests (group 1). Conditions not associated with vitamin D deficiency were detected in 57% (268/474) requests (group 2). A total cost of 25(OH)D testing for the CHIF was 58,729.50 EUR (25,523.79 EUR in the group 1 and 33,205.71 EUR in the group 2).

**Conclusions:**

More than half of all 25(OH)D tests performed in the clinical laboratory represent avoidable cost for the public healthcare. Prevention of population screening by vitamin D testing is needed.

## Introduction

According to a bibliometric analysis, a focus on vitamin D issues has been switched from musculoskeletal diseases in the early 2000s to endocrine diseases in the late 2000s with diabetes mellitus as the leading topic (*1*). Furthermore, an increase in the number of studies dedicated to the role of vitamin D in diverse clinical settings may mirror a simultaneous rise of vitamin D testing, mainly as 25-hydroxy vitamin D (25(OH)D) blood based test. On the other side, there has been a rising evidence of inappropriate use of 25(OH)D testing generating a heavy burden on healthcare system (*2*). In general, an inappropriate or unnecessary use of the laboratory test corresponds to the overtesting that may lead to the overdiagnosis causing that people with no signs and symptoms receive a medical condition (*3*). According to the definition, the overtesting may be detected by screening of the asymptomatic people by the non-recommended test or by performing more testing that is needed to diagnose patients with clinical conditions (*4*). One of the likely reasons for vitamin D extensive testing may lay in the resistance of the healthcare community and public to the recommendations of the medical professional associations that state against population screening for the vitamin D (*5*, *6*).

In 2016 several Croatian medical associations collaborated and published the guidelines for prevention, detection and therapy of vitamin D deficiency in adults (*6*).

In light of pandemic data of low vitamin D concentrations in the population, the guidelines state the recommendations for 25(OH)D testing for high-risk patients and advocate against population screening. The annual workload report in the Clinical Department for Laboratory diagnostics, Clinical Hospital Centre Rijeka revealed an increase of 36% in the 25(OH)D test orders in 2018. It made authors consider a trend of 25(OH)D overtesting that is potentially leading to overdiagnosis nevertheless of the statements in the current national guidelines.

We hypothesised that large number of 25(OH)D test orders were unnecessary and that those orders were avoidable cost for the Croatian National Health Insurance Fund (CHIF), a publicly funded healthcare fund. The aims of this study were an assessment of the number of the 25(OH)D tests requested in discordance with the national guidelines and an estimation of the related avoidable financial cost for public healthcare.

Due to the lack of the studies that evaluate an extent of the vitamin D overtesting in small population European countries with significant public funding of the healthcare, our findings may give useful insight in the topic.

## Materials and methods

### Methods

This study is a retrospective analysis of the anonymized patients’ data extracted from the Clinical Hospital Centre Rijeka information system database and the Clinical Department of Laboratory Diagnostics database, in the period between 1 January 2018 and 31 December 2018. Study protocol and data analyses were approved by the Hospital Ethics Committee (2170-29-02/1-19-2).

An initial analysis of extracted data (N = 5298) included records of all patients (adult and children, both genders) with at least one 25(OH)D test performed in the study period. The analysis included all requests for 25(OH)D testing from the hospital services (with the inpatient and outpatient care) and other healthcare services (out of the Clinical Hospital Centre Rijeka) in the Primorje-Gorski Kotar County. All the records from adult inpatients and children were excluded from further analysis (12%, 648/5298). The reason for the exclusion of adult’s inpatients records underlay in the policy of the CHIF that directly reimburses the cost of the individual test made for patients from the hospital outpatient care services and from other healthcare services (out of the hospital) but not for test performed for the hospital’s inpatients. Remaining data (N = 4650) were firstly stratified monthly across the studied year and then data of each month was randomised using Microsoft Office Excel (Microsoft, Washington, USA). First 10% of each month’s sorted data were taken into further analysis following a rationale that all randomised data had an equal potential for the analysis. An extraction of approximately 10% of records (474/4650) may reduce a workload and generate uncompromised results for analysis. If for some patient the data of the medical condition was not available in the hospital database, then the next one in line was included in the study. After that the randomized sample was checked for duplicate records and no such records were found. Finally, 474 extracted records were divided in two groups based on associated medical condition(s) checked in the hospital database. Group 1 included records with diagnosis and/or treatment that carried a high-risk for vitamin D deficiency (high-risk group). In contrast, group 2 included records with medical condition(s) not associated with risk for vitamin D deficiency (overtesting group). The classification of the medical condition(s) or treatment(s) in the two groups was based on the list of high-risk indicators for vitamin D deficiency from the Croatian guidelines for prevention, detection and therapy of vitamin D deficiency in adults. According to the guidelines, following conditions present high-risk indicators: osteoporosis, osteomalacia, rickets; malabsorption (cystic fibrosis, inflammatory bowel disease, untreated celiac disease, bariatric surgery, radiation enteritis); hyperparathyroidism, hypo- or hypercalcemia, hypophosphatemia; chronic kidney disease and kidney transplantation; liver failure; patients with high activity of alkaline phosphatase but normal function liver tests; medications: glucocorticoids, anticonvulsants, antifungals; obesity (body mass index, BMI > 30 kg/m^2^); older adults (> 50 years) with history of falls and fractures; pregnant and lactating women; granuloma-forming disorders (sarcoidosis, tuberculosis, histoplasmosis, coccidiomycosis, berylliosis); dark skin and decreased sun exposure; some lymphomas. If for one patient there were several diverse medical conditions or treatments, but at least one from the list, that record was eligible for classification in the group 1. No additional steps were done with the selected records.

All disputes regarding medical conditions and treatment classification were discussed and agreed between the authors of the study.

### Statistical analysis

#### Financial analysis - an estimation of 25(OH)D costs

A burden of 25(OH)D testing was calculated on basis of the price of single 25(OH)D test reimbursed by the CHIF. In 2018 the CHIF reimbursed to the CHC Rijeka 12.63 EUR for single 25(OH)D test performed by an immunoassay.

In an estimation of the 25(OH)D cost, it was assumed that a total number of the records from group 1 (high-risk group) corresponded to the number of 25(OH)D tests requested in accordance with the national guidelines and represented necessary cost for the national healthcare system. In contrast, the annual cost of the 25(OH)D testing in the group 2 (overtesting group) reflected avoidable cost for the public healthcare.

The total annual cost of vitamin D tests in all included records was calculated as following:

AC_TOT_ = N x P Equation (Eq.) 1.

AC_TOT_ – total annual cost

P – test price

N – total number of vitamin D tests (patients’ records) included in analysis (N = 4650).

The annual costs of 25(OH)D testing in the group 1 and group 2 were calculated according to following equations:

AC_G1_ = P x N_G1_ x (N / N_RR_) Eq. 2.

AC_G2_ = P x N_G2_ x (N / N_RR_) Eq. 3.

AC_G1_ – group 1 annual cost

P – test price

N – total number of vitamin D tests (patients’ records) included in analysis (N = 4650)

N_G1_ – number of vitamin D tests (patients’ records) in group 1 (N = 206)

N_RR_ – number of randomised vitamin D tests (patients’ records) (N = 474)

AC_G2_ – group 2 annual cost

N_G2_ - number of vitamin D tests (patients’ records) in group 2 (N = 268).

In both groups estimated costs were reported in EUR.

Financial analysis was done in the Microsoft Office Excel (Microsoft, Washington, USA).

## Results

Final sample of 474 extracted records included 381 (80%) female and 93 (20%) male patients, median age 61 (range 19–86) years.

The group 1 consisted of 206 (43%) patients with at least one medical condition or medication associated with high-risk for vitamin D deficiency according to the current national guidelines. Top three indicators for 25(OH)D testing were: i) osteoporosis (no rickets and osteomalacia cases) (79/206), ii) chronic kidney disease and kidney transplantation (43/206), and iii) obese adults (BMI > 30 kg/m^2^) (36/206). The frequencies of high-risk conditions or treatments for vitamin D deficiency found in our analysis for group 1 are presented in table 1.

**Table 1:** List of high-risk indicators for 25(OH)D testing and their frequencies in group 1 (N = 206)

**High-risk medical conditions or treatments**	**N (%)**
Osteoporosis	79 (38)
Chronic kidney disease and kidney transplantation	43 (21)
Obese adults (BMI > 30 kg/m^2^)	36 (18)
Older adults (> 50 years) with history of falls and fractures	21 (10)
Hyperparathyroidism	9 (4)
Medications (anticonvulsants, glucocorticoids)	8 (4)
Malabsorption syndrome (Inflammatory bowel disease)	6 (3)
Some lymphomas	3 (1.5)
Granuloma-forming diseases (tuberculosis)	1 (0.5)
Liver failure	0 (0)
Alkaline phosphatase high activity with normal function liver tests	0 (0)
Pregnant and lactating women	0 (0)
Dark skin/decreased sun exposure	0 (0)
25(OH)D – 25-hydroxy vitamin D. BMI – body mass index.	

In contrast to group 1, the information system database search found that for 268 (57%) records there was no evidence for an association with vitamin D deficiency (group 2).

The total cost of the 25(OH)D testing based on the number of records included in the study (N = 4650) was 58,729.50 EUR. A calculation of the 25(OH)D test expenditure in group 1 (N = 206) found that 2601.78 EUR were reimbursed by the CHIF. The recalculation of that charge based on all included records (N = 4650) made an annual sum of 25,523.79 EUR reimbursed by the CHIF for 25(OH)D tests. That sum can be considered as a necessary cost for a management of high-risk medical condition(s). Estimation of the 25(OH)D test price in overtesting group (group 2) (N = 268) revealed an expenditure of 3384.84 EUR which when recalculated on all records of 25(OH)D tests, gave a sum of 33,205.71 EUR. That implies that 57% (33,205.71/58,729.50 EUR) of all money that the CHIF reimbursed for 25(OH)D tests was spent in the overtesting group.

## Discussion

The present study revealed that more than half of performed vitamin D tests (57%) likely indicated avoidable expenditure for the public healthcare service. In financial terms inappropriate ordering of 25(OH)D test, may cost national public healthcare insurance fund 33,205.71 EUR annually.

A trend of inappropriate vitamin D testing seems to be resistant to the current guidelines as Norton *et al.* addressed in their study. They found that overall 52% in one Australian tertiary referral hospital were supported by the guidelines (*7*). Interestingly, the presented result roughly matches to our finding, but it may be worth to point out that both studies extrapolated data from the representative samples and excluded children population. Additionally, both studies found that top two indications supported by the national guidelines were osteoporosis and chronic kidney disease. However, in the Australian study obesity was excluded but in our study, it was top three high-risk indicators for vitamin D deficiency, making 18% of all requests supported by the guidelines. According to the official report of Croatian health profile in 2019, prevalence of obesity in Croatian adult population includes one obese person in five grown-ups (*8*). This can explain our study findings that the obesity is one of top three high-risk conditions for vitamin D deficiency.

Potential source of vitamin D excessive testing among clinicians may be a confounding data from diverse clinical guidelines as well as an information overload about vitamin D role in extra-skeletal diseases (malignancy, autoimmune disease, neurological diseases, and endocrine disease) associated with limited level of evidence (*7*). Unsurprisingly, it may put some pressure on the clinicians and patients, too. There is an opinion that patient expectation along with defensive medicine, lack of knowledge and profit are major reasons for overtesting (*4*). Finally, an individual clinician may order an extra test in context of patient’s medical condition and treatment out of current guidelines simply considering the patient benefit (*4*).

An observed rise of performed vitamin D tests associated with increased expenditure mostly has been reported as a cost that only included the reagent price (*9*). In contrast, in our study vitamin D testing costs in both groups as well as total cost include the reagent price along with personnel and extra personnel cost.

A potential of saving based on the vitamin D reagents cost was shown by Naugler *et al.* in a study that assessed an introduction of the Choosing Wisely Canada strategy in Alberta. The authors found 91% reduction of performed vitamin D tests with overall expenditure decrease of 1 million Canadian dollars or 684,930.00 EUR (*10*). The Choosing Wisely Canada campaign is an initiative supported by several medical associations that advocates a prevention of the vitamin D overtesting by a restriction of the test ordering on five medical conditions: metabolic bone disease, celiac malabsorption syndrome, chronic kidney disease, abnormal blood calcium and chronic liver disease (*11*).

Regarding limitations of our study, vitamin D test annual load in our clinical laboratory may be relatively modest in comparison to other tertiary health centre in Croatia (*12*). Another limitation may be an inevitable lack of a fullness of the medical data in the hospital and laboratory database according to which assessment of medical condition was made.

Further, in our study the records of all performed vitamin D tests for children and for adult inpatients from hospital wards and for from the healthcare services out of the hospital were excluded. Since, that records may make a substantial part in the total number of annually reported tests, their selected exclusion may carry on certain bias.

In conclusion, presented study indicates a need for more active approach in prevention of population screening by the 25(OH)D test as well as a potential for extensive financial saving for a public healthcare.
